# Synthesis and Evaluation of 2,4-Disubstituted Quinazoline Derivatives with Potent Anti-Angiogenesis Activities

**DOI:** 10.3390/molecules19078916

**Published:** 2014-06-26

**Authors:** Guangjin Yu, Zeng Li, Liang Tang, Qiru Xiong

**Affiliations:** 1 Department of Chirurgery, First Affiliated Hospital of Anhui Medical University, Hefei 230032, China; E-Mails: yusysu@163.com (G.Y.); tang@163.com (L.T.); 2 Department of Chemistry, Anhui Medical University, Hefei 230032, China; E-Mail: stronglz110732@163.com

**Keywords:** 2,4-disubstituted quinazoline derivatives, synthesis, anti-angiogenesis

## Abstract

A series of 2,4-disubstituted quinazoline derivatives were designed and synthesized. The biological results showed that most of quinazoline derivatives exhibited potent antiproliferative activities against a panel of three tumor cell lines and a good inhibitory effect against the adhesion and migration of human umbilical vein endothelial cells (HUVECs). Among these compounds, **11d** was the most potent agent, that alsoexhibited the highest anti-angiogenesis activities in the chick embryo chorioallantoic membrane (CAM) assay.

## 1. Introduction

Angiogenesis, the physiological process through which new blood vessels form from pre-existing vessels, often provides nutrients and oxygen to energetically proliferating tumor cells to support solid tumor growth [1]. Thus, it plays a key role in tumor progression and metastasis. Recent reports have been shown that without angiogenesis, a solid tumor can only reach the size of 1–2 mm^3^, which is small enough to be treated with conventional cytotoxic chemotherapeutic drugs [2]. In the past two decades, a number of angiogenesis inhibitors have been advanced into clinical development in cancer therapy, such as the fumagillin derivative TNP-470 [3], SU-5416 [4], angiostatin [5], and endostatin [6]. Therefore, the search for anti-cancer drugs targeting angiogenesis is a promising strategy for cancer therapy. 

**Figure 1:**
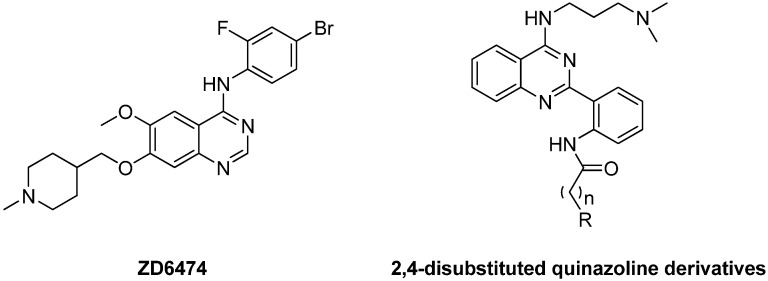
Structure of **ZD6474** and 2,4-disubstituted quinazoline derivatives.

Quinazolines, which are heterocyclic compounds consisting of two closed six-membered aromatic rings, represent an important class of scaffolds are found in a number of naturally occurring and synthetic molecules possessing a broad spectrum of biological activities. Astra Zeneca’s ZD6474 (vandetanib, Figure 1) is a novel heteroaromatic-substituted anilinoquinazoline that was discovered to be a potent and reversible inhibitor of VEGF receptor-2 (VEGFR-2), EGFR and RET tyrosine kinase (y) [7]. In preclinical trials, ZD6474 was shown to have broad spectrum antitumor activity in mouse models and was found to prevent the growth of tumors by inhibition of angiogenesis [8]. 

According to the structural features of ZD6474, we synthesized a series of 2,4-disubstituted quinazoline derivatives (Figure 1), hoping to screen for new and better inhibitors of angiogenesis. The approach was divided into two steps: (i) replacement of the C-4 anilino moiety of ZD6474 with a long side chain with a terminal amino group; (ii) introduction of another side chain such as an alkylaminoanilino side chain on the 2-position of the substituted aromatic group. This can significantly increase the ability of these compounds to form hydrogen bonds with the relevant desired receptors such as VEGFR-2, EGFR or RET tyrosine kinase. Their cytotoxicity against normal cell lines (human umbilical vein endothelial cell line, HUVEC) and tumor cell lines (CNE-2, PC-3, and SMMC-7721 cell lines) were evaluated. Furthermore, their anti-angiogenesis activities were examined by biophysical and biochemical assays, including cell adhesion, migration and chorioallantoic membrane of the chick (CAM) embryo assays.

## 2. Results and Discussion

### 2.1. Chemistry

**Scheme 1:**
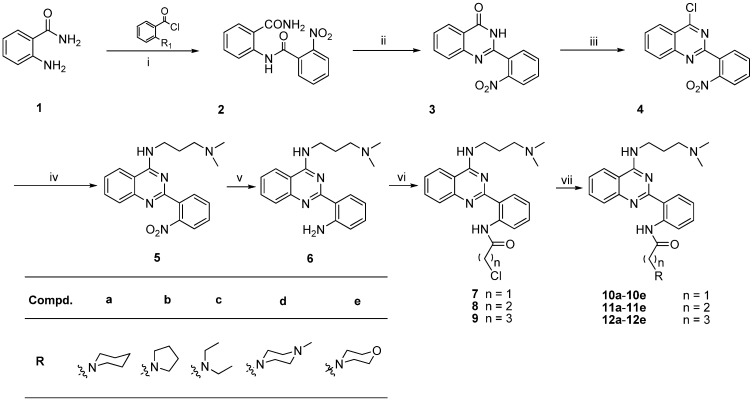
Synthesis of quinazoline derivatives.

The synthetic route to the quinazoline derivatives is outlined in Scheme 1. The uncyclized amide intermediate **2** was obtained by amidating 2-nitrobenzoyl chloride with anthranilamide in trichloromethane. The heterocyclic skeleton of quinazolinone **3** was formed through one step reaction of substrate **2**, with an oxidative ring closure reaction under basic conditions [9]. Then, treatment of the quinazolinone **3** with excess phosphorus oxychloride in refluxing toluene in the presence of *N,N*-diethylaniline gave compound **4**. Coupling of 3-(dimethylamino)-1-propylamine with quinazoline intermediate **4** in THF at 66 °C, afforded compound **5**. In order to obtain the amine functionality, compound **6** was prepared by treatment of nitro-substituted compound **5** with 80% hydrazine hydrate and 10% Pd/C in isopropanol. Treatment of the amine-substituted compound **6** with acyl chlorides and potassium carbonate in dichloromethane gave compounds **7**–**9** at 69%–85% yield. Finally, the target compounds **10a**–**i**, **11a**–**e**, **12a**–**e** were obtained through aminolysis of the compounds **7**, **8**, and **9** under reflux through the treatment with the appropriate secondary amines.

*Reagents and Conditions*: (i) 2 equiv. of TEA, CHCl_3_, rt, 5 h; (ii) 10% aqueous KOH, EtOH, reflux, 2 h; (iii) *N*,*N*-diethylaniline, POCl_3_, toluene, reflux, 6 h; (iv) 3-aminopropyldimethyl- amine, THF, reflux, 5 h; (v) 10% Pd/C, 80% N_2_H_4_^.^H_2_O, isopropanol, reflux, 2 h; (vi) Cl(CH_2_)_n_COCl, K_2_CO_3_, CH_2_Cl_2_, rt, 24 h; (vii) R_2_NH, KI, ethanol, reflux.

### 2.2. Cytotoxicity Against Tumor Cells and HUVEC

In the early 80s, scientists proposed anti-angiogenesis as a potential target in cancer therapy [10]. Much research in the past 30 years has targeted tumor angiogenesis as a means of inhibiting the process of tumor-caused blood vessel formation. Endothelial cells, which come from the inner lining of blood vessels, makes up a fundamental part of new blood vessels as well as pre-existing ones. A complete angiogenesis process includes the proliferation, migration, and differentiation of endothelial cells, so a great deal of angiogenesis inhibitors discovered to date target endothelial cells. We thus employed a cell proliferation assay to evaluate the cytotoxicity of quinazoline derivatives against both normal (HUVEC) and tumor cell lines (CNE-2, PC-3, and SMMC-7721 cell lines) so we can screen some molecules that have cytotoxicity against tumor cells but low cytotoxicity against HUVECs. Meanwhile, an appropriate concentration range of these compounds could be determined to further study their inhibitive effects against the proliferation, migration, and differentiation of HUVECs. The cytotoxic activities of quinazoline derivatives against human tumor cell lines CNE-2 (human nasopharyngeal cancer), PC-3 (human prostatic carcinoma), SMMC-7721 (human liver cancer) and Human Umbilical Vein Endothelial Cells (HUVECs) were determined by using the MTT cytotoxicity assay. The results showed that most of the 2,4-disubstituted quinazoline derivatives possessed high cytotoxicity against human tumor cell lines and moderate cytotoxicity against HUVECs. Table 1 showed the IC_50_ values (cytotoxicity potency indexes) of all the derivatives against four types of cells lines. 

**Table 1:** IC_50_ cytotoxicity values (µM) of the quinazoline derivatives against tumor cells and HUVEC.

	IC_50_ (μM)					IC_50_ (μM)				
ompd	CNE-2	PC-3	7721	HUVEC	Compd	CNE-2	PC-3	7721	HUVEC	
**10a**	13.6 ± 0.2	16.8 ± 0.1	18.1 ± 0.2	45.7 ± 0.2	**11d**	9.3 ± 0.2	9.8 ± 0.3	10.9 ± 0.2	47.2 ± 0.2
**10b**	14.9± 0.2	16.7 ± 0.2	17.9 ± 0.4	39.3 ± 0.3	**11e**	20.1 ± 0.2	19.6 ± 0.2	20.9 ± 0.3	84.9 ± 0.3
**10c**	17.1 ± 0.4	17.6 ± 0.1	19.4 ± 0.1	56.2 ± 0.2	**12a**	17.3 ± 0.3	17.7 ± 0.2	16.5 ± 0.1	41.7 ± 0.3
**10d**	10.5 ± 0.3	11.3 ± 0.5	10.4 ± 0.1	38.7 ± 0.1	**12b**	16.4 ± 0.3	18.9 ± 0.4	15.3 ± 0.2	39.4 ± 0.1
**10e**	19.7 ± 0.2	20.5 ± 0.3	22.6 ± 0.2	79.8 ± 0.1	**12c**	19.9 ± 0.2	17.1 ± 0.3	17.4 ± 0.1	44.5 ± 0.2
**11a**	14.2 ± 0.1	18.1 ± 0.2	19.9 ± 0.4	57.4 ± 0.5	**12d**	11.4 ± 0.1	10.9 ± 0.2	10.3 ± 0.5	40.2 ± 0.2
**11b**	14.3 ± 0.1	17.4 ± 0.1	15.1 ± 0.2	46.3 ± 0.3	**12e**	20.6 ± 0.2	21.9 ± 0.2	20.8 ± 0.3	76.3 ± 0.2
**11c**	16.8 ± 0.4	18.7 ± 0.3	13.8 ± 0.1	66.8 ± 0.3					

Data derived from the mean of three independent assays.

The results indicate that the IC_50_ values on the tested tumor cells are in the range of 10–40 µM. Among all the derivatives, **11d** was the most potent in inhibiting the tumor cell proliferation with the lowest IC_50_ values of 9.3 ± 0.2 μM (CNE-2), 9.8 ± 0.3 μM (PC-3) and 10.9 ± 0.2 μM (SMMC-7721), respectively. Compound **10e** exhibited the lowest cytotoxicity to the various cancer cells lines. Comparing with quinazoline derivatives **11d**, **12d** and **10d**, which had the same R group, it was found compound **10d** was much less cytotoxic against HUVECs than **11d** and **12d**, although they had comparative cytotoxicity against tumor cells. As for the structure-activity relationships, the n = 2 amide side chain analogues of compounds (**11** series) show the highest cytotoxicity against human tumor cell lines, while no significant effect on IC_50_ values was observed with the extension of the amide side chain (n > 2). On the other hand, alterations of the basic terminus of the amide side chains show that the *N*-methylpiperazino analogues (**10d**, **11d** and **12d**) had the most enhanced cytotoxicity for human tumor cell lines compared to other haloalkanamido-substituted compounds. All the above results showed that the cytotoxicity of these quinazoline derivatives was closely related to their R group and the length of the side chains. 

### 2.3. Inhibition of HUVEC Adhesion

A perfect angiogenesis inhibitor must be show lower toxicity toward HUVECs, while showing high inhibitive capability towards the proliferation, migration, and differentiation of HUVECs. To assess the inhibitory effects of 2,4-disubstituted quinazoline derivatives, three assays were used: HUVEC adhesion assay, HUVEC migration assay and CAM assay. Firstly, to test the inhibitory effects of quinazoline derivatives on the attachment of endothelial cells to type I collagen, cell adhesion assays were carried out. 

As shown in Table 2, most compounds inhibited HUVEC adhesion to collagen. Most of the quinazoline derivatives significantly inhibited HUVEC adhesion at both 1 h and 3 h. Compound **11d** showed the most potent inhibition of HUVEC adhesion, with an inhibitory rate of as much as 65.8% ± 0.2% at a dose of 15 μM and 71.2% ± 0.1% at a dose of 30 μM after 3 h incubation at 37 °C. Compound **12d** showed similar results with **11d** for the experiment, while **10d**, with the shortest amide side chains and the same basic terminus as **11d** showed an average inhibition of HUVEC adhesion, with a inhibitory rate of 63.7% ± 0.2% at a dose of 15 µM and 63.4% ± 0.3% at a dose of 30 µM when 3 h incubation at 37 °C. Alterations of the basic terminus of the amide side chains show that the *N*-methylpiperazino analogues had strongly enhanced inhibition of HUVEC adhesion compared to the piperidino, pyrrolidino and diethylamino analogues, with the less basic morpholino analogues proving favorable to HUVEC adhesion. These results demonstrated the importance of then amide side chain length and basicity for potent inhibition of HUVEC adhesion. 

**Table 2:** The inhibitory effects of compounds on adhesion of HUVEC on collagen at a dose of 15 μM and 30 μM.

Compd.	1 h Inhibition Rate %		3 h Inhibition Rate %	
	Dose 15 μM	Dose 30 μM	Dose 15 μM	Dose 30 μM
**10a**	26.7 ± 0.1	31.6 ± 0.3	29.2 ± 0.2	37.6 ± 0.1
**10b**	28.3±0.1	33.0 ± 0.2	27.8 ± 0.2	34.8 ± 0.2
**10c**	18.9 ± 0.3	27.8 ± 0.2	16.4 ± 0.4	29.5 ± 0.1
**10d**	34.8 ± 0.2	44.6 ± 0.4	32.5 ± 0.4	47.2 ± 0.1
**10e**	nd	14.3 ± 0.1	7.9 ± 0.3	17.6 ± 0.3
**11a**	37.4 ± 0.3	49.6 ± 0.5	38.2 ± 0.1	59.3 ± 0.1
**11b**	35.4 ± 0.3	54.3 ± 0.3	35.2 ± 0.3	51.7 ± 0.2
**11c**	29.9 ± 0.2	38.7 ± 0.2	29.4 ± 0.2	36.3 ± 0.4
**11d**	47.4 ± 0.1	65.8 ± 0.2	53.3 ± 0.3	71.2 ± 0.1
**11e**	27.4 ± 0.3	32.3 ± 0.2	25.6 ± 0.3	36.4 ± 0.3
**12a**	33.6 ± 0.2	57.9 ± 0.3	40.6 ± 0.3	61.2 ± 0.3
**12b**	29.8 ± 0.1	49.3 ± 0.1	38.4 ± 0.2	60.1 ± 0.2
**12c**	24.2 ± 0.3	44.3 ± 0.2	27.9 ± 0.2	49.5 ± 0.2
**12d**	46.8 ± 0.2	63.7 ± 0.2	47.5 ± 0.3	63.4 ± 0.3
**12e**	26.5 ± 0.2	34.7 ± 0.2	17.2 ± 0.4	39.4 ± 0.2

Data derived from the mean of three independent assays; nd means no results due to no inhibition.

### 2.4. Inhibition of HUVEC Migration

**Figure 2:**
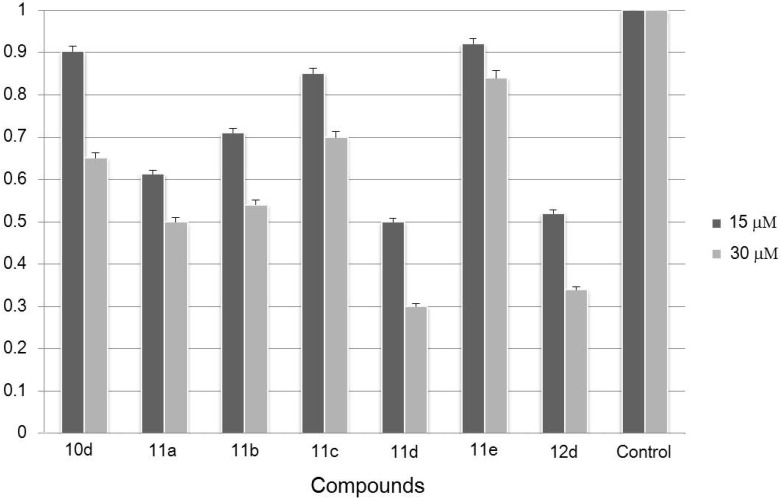
The inhibitory effects of quinazoline derivatives on HUVEC migration at doses of 15 μM and 30 μM.

According to the HUVEC adhesion assay results in which most of the 2,4-disubstituted quinazoline derivatives showed better inhibitory effects against HUVEC adhesion, compounds **10d**, **11a**–**e**, **12d** were chosen to evaluate their inhibition against HUVEC migration in a HUVEC wounding assay which is an important procedure in tube formation and vessel sprouting. Compounds were tested at concentrations of both 15 μM and 30 μM, and all seven compounds tested in this assay could inhibit HUVEC migration. Five derivatives, **11a**–**e**, with a longer side chain in the 2-position of the parent structure C, showed a greatly inhibitory potential on HUVEC migration at 15 μM (Figure 2), and the inhibitory effects were more significant when the concentration of these compounds was increased to 30 μM. Among all these compounds, **11d** showed the best inhibitory activity. As for the structure-activity relationship, the results from these migration experiments suggest that quinazoline derivatives bearing the longer amide chains (n ≥ 2) should have potent inhibitory activity against HUVEC migration, which further supported the results of the adhesion studies indicating the importance of amide side chain length.

### 2.5. 2,4-Disubstituted Quinazoline Derivatives Inhibits Angiogenesis in Vivo in the CAM Assay

**Figure 3:**
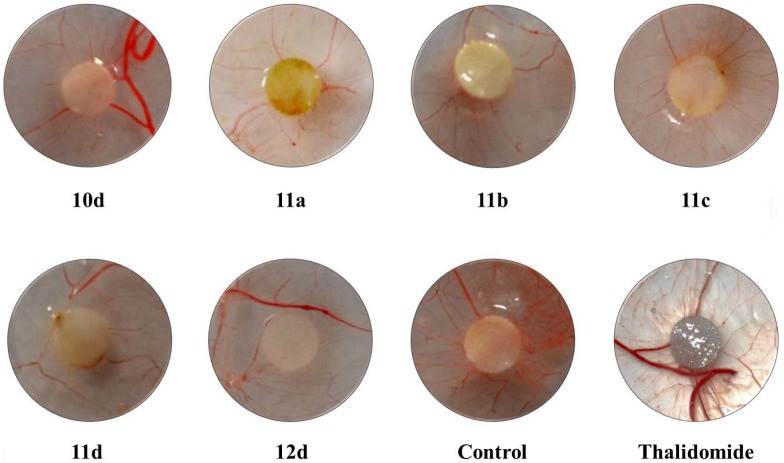
The results of CAM assay. NaCl solution (0.9%) and thalidomide were used as a negative control and a positive control, respectively. Representative pictures of independent experiments performed in duplicate for the quinazoline derivatives **10d**, **11a**–**d** and **12d** doses and controls. All filter discs had the same size, and any visual size differences are due to the various distances from which pictures were taken.

Based on the HUVEC cytotoxicity, migration, and adhesion assay results, some 2,4-disubstituted quinazoline derivatives (compounds **10d**, **11a**–**d**, **12d**) possessing moderate cytotoxicity and potent inhibitive effect against the adhesion and migration of HUVEC, were chosen to assess their anti-angiogenesis activities by an *in vivo* CAM assay. CAMs treated with 0.9% NaCl solution (negative control) were surrounded by all antoic vessels as newly-formed capillaries converging radially toward the sponge in a “spoked wheel” pattern (number of vessels 24 ± 3). As shown in Figure 3, there was a significant reduction of the angiogenic response when quinazoline derivatives were added to the CAMs. Among all CAMs treated with 2,4-disubstituted quinazoline derivatives, compounds **11d**, **12d** show excellent inhibition of angiogenesis in chick embryos, which further supports the cytotoxicity, adhesion and migration results. A comparison of the CAM assay results with the reference thalidomide (a clinical anti-tumor drug targeting tumor angiogenesis) and all the compounds tested revealed that these quinazoline derivatives show better inhibition of angiogenesis than thalidomide [11].

## 3. Experimental

### 3.1. General Information

^1^H-NMR (400 MHz) and ^13^C-NMR (101 MHz) spectra were recorded on a Bruker 400 NMR instrument with tetramethylsilane (TMS) as an internal standard. Only new compounds’ NMR data is given. MS spectra were obtained using a Shimadzu LC-MS-2010A spectrometer. Melting points were determined on an X-6 microscope melting point instrument and are uncorrected.

*N-[2-(Aminocarbonyl)phenyl]-2-nitrobenzamide* (**2**). 2-Nitrobenzoic acid (11.04 g, 54.9 mmol) and thionyl chloride (50 mL) were mixed and the reaction mixture was refluxed for 1.5 h at 80 °C. The solution was allowed to cool at room temperature, followed by the evaporation of excess thionyl chloride *in vacuo*. The resulting wine red solution was taken and added dropwise to a solution of anthranilamide (**1**; 9.36 g, 76.6 mmol) and triethylamine (27.9 mL, 153.2 mmol) in chloroform (200 mL) and stirred at room temperature for 5 h. The precipitated solid was collected by filtration, washed with ethanol, and dried. Recrystallization from ethanol afforded the product **2** (15.1 g, 86%) as a white solid; mp 219–221 °C. ^1^H-NMR (DMSO-d_6_) *δ =* 12.56 (s, 1H), 8.44 (d, *J* = 8.2 Hz, 1H), 8.39 (s, 1H), 8.26 (d, *J* = 1.6 Hz, 1H), 8.04–7.96 (m, 1H), 7.93–7.85 (m, 2H), 7.81 (s, 1H), 7.59 (t, *J* = 7.8 Hz, 1H), 7.24 (t, *J* = 7.6 Hz, 1H). ^13^C-NMR (DMSO-d_6_) *δ =* 170.63, 162.27, 148.00, 138.95, 135.72, 133.61, 132.35, 130.45, 129.92, 128.68, 124.56, 123.52, 120.63, 120.38. ESI-MS *m/z*: 320 [M+H]^+^.

*2-(2-Nitrophenyl)-3H-quinazolin-4-one* (**3**). A mixture of benzamide **2** (12 g, 37.6 mmol) in 10% aqueous KOH (100 mL) and EtOH (100 mL) was heated to reflux for 2 h. Ethanol was removed *in vacuo*, and the aqueous layer was acidified with hydrochloric acid to pH 3 to give a solid residue. The solid was filtered and washed with water, which was purified by column chromatography, eluting with petroleum ether/EtOAc (3:1) to furnish pure **3** (11.2 g, 92%) as a white solid; mp 263–264 °C.^ 1^H-NMR (DMSO-d_6_) *δ* = 12.87 (s, 1H), 8.19 (d, *J* = 2.0 Hz, 1H), 8.05 (d, *J* = 8.0 Hz, 1H), 7.89 (dd, *J* = 8.4, 2.0 Hz, 1H), 7.78 (d, *J* = 8.4 Hz, 1H), 7.71 (t, *J* = 7.8 Hz, 1H), 7.51 (d, *J* = 8.2 Hz, 1H), 7.44 (t, *J* = 7.7 Hz, 1H). ^13^C-NMR (DMSO-d_6_) *δ =* 161.46, 150.60, 148.30, 148.18, 135.66, 134.55, 133.53, 132.99, 127.70, 127.26, 127.15, 125.86, 124.41, 121.19. ESI-MS *m/z:* 302 [M+H]^+^.

*2-(4-Chloro-2-nitrophenyl)quinazoline* (**4**). To a mechanically stirred suspension of **3** (10 g, 33.2 mmol) in toluene (100 mL) was added *N,N-*diethylaniline (8,2 g, 55.2 mmol), followed by phosphorus oxychloride (6.1 g, 39.8 mmol). The reaction was then heated to 80 °C for 6 h, allowed to cool then washed with iced water (200 mL). The organic layer was then washed sequentially with 20% NaOH (2 × 100 mL), iced water (200 mL) and brine (200 mL) and dried over anhydrous sodium sulfate. The organic solvent was removed in vacuo and the residue was purified by column chromatography with CHCl_3_/CH_3_OH (15:1) to give **4** (5.1 g, 48%) as a colorless powder. The aqueous layers were combined and extracted with dichloromethane. The organic layer was reduced *in vacuo* to yield a yellow solid, which was purified by column chromatography as above, providing further product to give a total yield of 6.5 g (61%). mp 189–191 °C. ^1^H-NMR (DMSO-d_6_) *δ =* 8.36–8.31 (m, 1H), 8.26 (d, *J* = 2.1 Hz, 1H), 8.23–8.17 (m, 2H), 8.10 (d, *J* = 8.1 Hz, 1H), 7.96 (ddd, *J* = 9.1, 5.9, 2.2 Hz, 2H). ^13^C-NMR (CDCl_3_) *δ =* 162.69, 157.00, 151.29, 150.32, 136.63, 135.33, 132.94, 132.06, 130.16, 129.47, 129.00, 125.82, 124.35, 122.50. ESI-MS *m/z*: 321 [M+H]^+^.

*N1-(2-(2-Nitrophenyl)quinazolin-4-yl)-N3,N3-dimethylpropane-1,3-diamine* (**5**). To a suspension of **4** (5 g, 15.7 mmol) in dried THF (100 mL) was slowly added dried 3-(dimethylamino)-1-propylamine (5.9 mL, 47.1 mmol) under a N_2_ atmosphere. The mixture was refluxed for 5 h, at which point TLC analysis indicated reaction completion. After concentration *in vacuo*, the resulting residue was dissolved in CHCl_3_, washed with water and brine, dried over anhydrous MgSO_4_ and evaporated. The residue was chromatographed on silica gel eluting with CHCl_3_/CH_3_OH (30:1) to give **5** (3.1 g, 51%) as a white solid. mp 179–181 °C. ^1^H-NMR (CDCl_3_) *δ =* 8.95 (s, 1H), 8.21 (d, *J* = 8.4 Hz, 1H), 7.84 (d, *J* = 8.3 Hz, 1H), 7.75–7.66 (m, 2H), 7.62–7.56 (m, 2H), 7.45 (t, *J* = 7.3 Hz, 1H), 3.66 (dd, *J* = 10.4, 5.6 Hz, 2H), 2.63–2.55 (m, 2H), 2.38 (s, 6H), 1.88–1.82 (m, 2H).^ 13^C-NMR (CDCl_3_) *δ =* 159.58, 158.07, 150.86, 149.87, 135.18, 132.62, 132.51, 132.45, 131.33, 128.50, 126.17, 123.53, 121.14, 114.15, 59.46, 45.36, 42.16, 24.64. ESI-MS *m/z*: 386 [M+H]^+^.

*N1-(2-(2-Aminophenyl)quinazolin-4-yl)-N3,N3-dimethylpropane-1,3-diamine* (**6**). A mixture of compound **5** (10 g, 26 mmol) in isopropanol (100 mL) and 80% hydrazine hydrate (40 mL) was added to a suspension of 10% Pd/C (1 g) in chilled isopropanol (30 mL), and the suspension was refluxed for 2 h. After the reaction was complete, the mixture was filtered through a pad of Celite, washed with isopropanol and then concentrated *in vacuo*. The residue was then suspended in 5% NH_3(aq)_ solution (100 mL). The solution was then extracted with EtOAc (3 × 100 mL), the organics dried over anhydrous Na_2_SO_4_ and then removed in vacuo. The solid obtained was dried under vacuum to give **6** (8.7 g, 96%) as a white powder. mp 182–183 °C. ^1^H-NMR (CDCl_3_) *δ =* 8.57 (s, 1H), 8.51 (d, *J* = 8.7 Hz, 1H), 7.78–7.73 (m, 1H), 7.70–7.61 (m, 2H), 7.41–7.35 (m, 1H), 6.77 (s, 2H), 6.71 (dt, *J* = 8.3, 1.9 Hz, 2H), 3.84 (dd, *J* = 10.5, 5.8 Hz, 2H), 2.69–2.62 (m, 2H), 2.42 (s, 6H), 1.96 (dd, *J* = 6.7, 4.9 Hz, 2H).^ 13^C-NMR (CDCl_3_) *δ =* 161.56, 158.98, 149.96, 149.33, 136.28, 132.59, 132.12, 127.96, 125.05, 120.95, 118.78, 116.51, 115.96, 113.71, 59.72, 45.48, 42.44, 24.76. ESI-MS *m/z*: 356 [M+H]^+^.

*General acylation procedure.* A solution of the appropriate acid halide (9.6 mmol) in CH_2_Cl_2_ (10 mL) was added during to a well-stirred mixture of the amino-substituted compound **6** (2.6 g, 8 mmol) and K_2_CO_3_ (1.6 g) in CH_2_Cl_2_ (50 mL) at 0 °C in a ice bath. The reaction was then allowed to warm up to RT, then stirred overnight. After cooling to 0 °C, the precipitate formed was filtered off and was further purified by flash column chromatography with petroleum ether/EtOAc (10:1) elution to give the compounds **7**–**9**.

*2-Chloro-N-(2-(4-(3-(dimethylamino)propylamino)quinazolin-2-yl)phenyl)acetamide* (**7**). Compound **6** was treated with chloroacetyl chloride according to general acylation procedure to afford **7** as a white solid in 85% yield; mp 227–228 °C. ^1^H-NMR (CDCl_3_) *δ =* 14.29 (s, 1H), 8.84 (s, 1H), 8.81 (d, *J* = 2.1 Hz, 1H), 8.62 (d, *J* = 8.6 Hz, 1H), 7.95 (d, *J* = 8.7 Hz, 1H), 7.84 (d, *J* = 8.3 Hz, 1H), 7.73 (ddd, *J* = 8.3, 7.0, 1.3 Hz, 1H), 7.50–7.44 (m, 1H), 7.17 (dd, *J* = 8.6, 2.2 Hz, 1H), 4.29 (s, 2H), 3.92 (dd, *J* = 11.0, 5.4 Hz, 2H), 2.86–2.76 (m, 2H), 2.55 (s, 6H), 2.09–2.02 (m, 2H).^ 13^C-NMR (CDCl_3_) *δ =* 165.43, 160.27, 159.32, 148.24, 139.93, 136.52, 132.56, 131.94, 127.54, 125.95, 123.37, 121.10, 120.39, 113.92, 59.82, 45.44, 43.79, 42.62, 24.47. ESI-MS *m/z*: 432 [M+H]^+^.

*3-Chloro-N-(2-(4-(3-(dimethylamino)propylamino)quinazolin-2-yl)phenyl)propanamide* (**8**). Using the general acylation procedure compound **6** was treated with 3-chloropropanoyl chloride to afford **8** as a white solid in 73% yield; mp 181–183 °C. ^1^H-NMR (CDCl_3_) *δ* 14.20 (s, 1H), 8.95 (s, 1H), 8.73 (d, *J* = 1.9 Hz, 1H), 7.73 (d, *J* = 3.9 Hz, 2H), 7.62 (d, *J* = 8.1 Hz, 1H), 7.47–7.41 (m, 2H), 7.17 (d, *J* = 8.3 Hz, 1H), 3.97 (t, *J* = 6.9 Hz, 2H), 3.86 (dd, *J* = 10.3, 5.5 Hz, 2H), 3.01 (t, *J* = 6.9 Hz, 2H), 2.65–2.61 (m, 2H), 2.40 (s, 6H), 1.95–1.88 (m, 2H). ^13^C-NMR (DMSO-d_6_) *δ* 169.96, 160.30, 159.12, 147.56, 139.90, 133.13, 130.85, 130.41, 126.81, 126.00, 122.97, 122.73, 122.17, 119.57, 113.31, 56.93, 45.00, 44.80, 34.76, 28.14, 26.12. ESI-MS *m/z*: 446 [M+H]^+^.

*4-Chloro-N-(2-(4-(3-(dimethylamino)propylamino)quinazolin-2-yl)phenyl)butanamide* (**9**). Compound **6** was treated with 4-chlorobutyryl chloride according to the general acylation procedure to afford **9** as a white solid in 69% yield; mp 156–157 °C. ^1^H-NMR (CDCl_3_) *δ =* 14.20 (s, 1H), 8.94 (s, 1H), 8.85 (d, *J* = 2.2 Hz, 1H), 8.67 (d, *J* = 8.6 Hz, 1H), 7.79–7.69 (m, 3H), 7.50–7.44 (m, 1H), 7.11 (dd, *J* = 8.6, 2.2 Hz, 1H), 3.90 (dd, *J* = 10.5, 5.7 Hz, 2H), 3.72 (t, *J* = 6.2 Hz, 2H), 2.77 (t, *J* = 5.0 Hz, 2H), 2.74 (d, *J* = 6.9 Hz, 2H), 2.49 (s, 6H), 2.30 (dt, *J* = 13.4, 6.6 Hz, 2H), 2.04–1.98 (m, 2H). ^13^C-NMR (CDCl_3_) *δ =* 170.56, 160.34, 159.00, 147.86, 141.09, 136.62, 132.59, 131.77, 127.30, 125.93, 122.34, 121.68, 121.20, 119.78, 113.83, 59.77, 44.66, 42.70, 35.29, 28.29, 24.45. ESI-MS *m/z*: 460 [M+H]^+^

*General aminolysis procedure.* To a stirred refluxing suspension of the chloride compounds **7**, **8** or **9** (0.5 mmol) and KI (0.08 g) in EtOH (15 mL) was added dropwise the appropriate secondary amine (2.0 mL) in EtOH (5 mL). The mixture was stirred at reflux for 5 h, cooled to 0 °C, then diluted with distilled water, filtered, and washed with ether and water, then evaporated under vacuum. The crude solid was purified by chromatography with petroleum ether/ EtOAc elution to afford **10a**–**e**, **11a**–**e** and **12a**, **12d**, **12e**.

*N-(2-(4-(3-(Dimethylamino)propylamino)quinazolin-2-yl)phenyl)-2-(piperidin-1-yl)acetamide* (**10a**). Compound **7** was treated with excess piperidine according to the general aminolysis procedure to afford **10a** as a white solid in 78% yield after column chromatography with petroleum ether/EtOAc (20:1) elution; mp 180–182 °C. ^1^H-NMR (CDCl_3_) *δ =* 13.51 (s, 1H), 9.00 (s, 1H), 8.89 (d, *J* = 2.1 Hz, 1H), 8.51 (d, *J* = 8.6 Hz, 1H), 7.99 (d, *J* = 8.2 Hz, 1H), 7.72 (t, *J* = 7.6 Hz, 1H), 7.60 (d, J = 8.0 Hz, 1H), 7.45 (t, *J* = 7.4 Hz, 1H), 7.12 (dd, *J* = 8.6, 2.1 Hz, 1H), 3.86 (dd, *J* = 10.2, 5.6 Hz, 2H), 3.26 (s, 2H), 2.67–2.61 (m, 2H), 2.57–2.44 (m, 4H), 2.41 (s, 6H), 1.94–1.87 (m, 2H), 1.46 (dt, *J* = 10.9, 5.6 Hz, 4H), 1.37–1.29 (m, 2H). ^13^C-NMR (CDCl_3_) *δ =* 170.12, 160.56, 159.41, 148.66, 140.14, 136.35, 132.17, 131.77, 128.41, 125.85, 123.76, 122.69, 121.02, 120.66, 113.84, 65.22, 59.93, 54.81, 45.50, 42.73, 25.28, 24.45, 23.84. HRMS (ESI): Calcd for (M−H)^−^ (C_26_H_33_ClN_6_O) requires *m/z* 479.2326, found 479.2320. Anal. Calcd for C_26_H_33_ClN_6_O^.^H_2_O: C, 62.57; H, 7.07; N, 16.84. Found: C, 62.46; H, 7.03; N, 16.84.

*N-(2-(4-(3-(dimethylamino)propylamino)quinazolin-2-yl)phenyl)-2-(pyrrolidin-1-yl)acetamide* (**10b**). Compound **7** was treated with excess pyrrolidine according to the general aminolysis procedure to afford **10b** as a white solid in 74% yield after column chromatography with petroleum ether/EtOAc (20:1) elution; mp 174–175 °C. ^1^H-NMR (CDCl_3_) *δ =* 13.84 (s, 1H), 8.93 (s, 2H), 8.57 (d, *J* = 8.1 Hz, 1H), 7.92 (d, *J* = 7.8 Hz, 1H), 7.69 (d, *J* = 6.6 Hz, 1H), 7.60 (d, *J* = 7.5 Hz, 1H), 7.43 (t, *J* = 6.9 Hz, 1H), 7.12 (d, *J* = 8.1 Hz, 1H), 3.86 (dd, *J* = 10.0, 5.8 Hz, 2H), 3.43 (s, 2H), 2.68 (t, *J* = 7.0 Hz, 4H), 2.65–2.52 (m, 2H), 2.40 (s, 6H), 1.97–1.84 (m, 2H), 1.71 (t, *J* = 7.2 Hz, 4H).^ 13^C-NMR (CDCl_3_) *δ =* 170.55, 160.50, 159.38, 148.72, 140.36, 136.42, 131.99, 131.76, 128.01, 125.79, 123.54, 122.66, 121.01, 120.69, 113.80, 62.22, 59.78, 54.52, 45.44, 42.53, 24.51, 23.93. HRMS (ESI): Calcd for (M−H)^−^ (C_25_H_31_ClN_6_O) requires *m/z* 465.2170, found 465.2162. Anal. Calcd for C_25_H_31_ClN_6_O^.^H_2_O: C, 61.91; H, 6.86; N, 17.33. Found: C, 61.88; H, 6.53; N, 17.38.

*N-(2-(4-(3-(Dimethylamino)propylamino)quinazolin-2-yl)phenyl)-2-(diethylamino)acetamide* (**10c**). Compound **7** was treated with excess diethylamine according to the general aminolysis procedure to afford **10c** as a white solid in 81% yield after column chromatography with petroleum ether/EtOAc (20:1) elution; mp 10–172 °C ^1^H-NMR (CDCl_3_) *δ =* 13.60 (s, 1H), 8.92 (d, *J* = 2.1 Hz, 2H), 8.53 (d, *J* = 8.6 Hz, 1H), 7.90 (d, *J* = 8.3 Hz, 1H), 7.71 (t, *J* = 7.3 Hz, 1H), 7.60 (d, *J* = 8.0 Hz, 1H), 7.43 (t, *J* = 7.5 Hz, 1H), 7.13 (dd, *J* = 8.6, 2.1 Hz, 1H), 3.86 (dd, *J* = 10.3, 5.5 Hz, 2H), 3.30 (s, 2H), 2.68 (q, *J* = 7.2 Hz, 4H), 2.65–2.55 (m, 2H), 2.40 (s, 6H), 1.90 (dd, *J* = 16.7, 5.9 Hz, 2H), 0.99 (t, *J* = 7.1 Hz, 6H).^ 13^C-NMR (CDCl_3_) *δ* = 172.03, 160.53, 159.44, 148.88, 140.01, 136.26, 132.04, 131.93, 128.02, 125.71, 124.22, 122.73, 121.02, 120.86, 113.85, 59.92, 58.81, 49.09, 45.51, 42.65, 24.53, 11.64. HRMS (ESI): Calcd for (M−H)^−^ (C_25_H_33_ClN_6_O) requires *m/z* 467.2326, found 467.2319. Anal. Calcd for C_25_H_33_ClN_6_O^.^H_2_O: C, 61.65; H, 7.24; N, 17.26. Found: C, 61.58; H, 7.21; N, 17.04.

*N-(2-(4-(3-(Dimethylamino)propylamino)quinazolin-2-yl)phenyl)-2-(4-methylpiperazin-1-yl)acetamide* (**10d**). Compound **7** was treated with excess *N*-methylpiperazine according to the general aminolysis procedure to afford **10d** as a white solid in 66% yield after column chromatography with petroleum ether/EtOAc (15:1) elution; mp 174–175 °C. ^1^H-NMR (DCl_3_) *δ =* 13.51 (s, 1H), 8.98 (s, 1H), 8.86 (d, *J* = 2.1 Hz, 1H), 8.49 (d, *J* = 8.6 Hz, 1H), 7.95 (d, *J* = 8.2 Hz, 1H), 7.77–7.67 (m, 2H), 7.47 (t, *J* = 7.5 Hz, 1H), 7.13 (dd, *J* = 8.6, 2.1 Hz, 1H), 3.87 (dd, *J* = 10.4, 5.5 Hz, 2H), 3.31 (s, 2H), 2.72–2.67 (m, 2H), 2.67–2.51 (m, 4H), 2.46 (s, 6H), 2.40–2.24 (m, 4H), 2.12 (s, 3H), 2.01–1.91 (m, 2H). ^13^C-NMR (CDCl_3_) *δ =* 169.36, 160.52, 159.45, 148.55, 139.93, 136.37, 132.42, 131.75, 128.46, 126.01, 123.79, 122.85, 121.29, 120.67, 113.86, 64.20, 59.29, 54.15, 53.35, 45.80, 45.20, 42.04, 24.31. HRMS (ESI): Calcd for (M−H)^− ^(C_26_H_34_ClN_7_O) requires *m/z* 494.2435, found 494.2421. Anal. Calcd for C_26_H_34_ClN_7_O^.^H_2_O: C, 60.75; H, 7.06; N, 19.07. Found: C, 60.66; H, 6.97; N, 18.89.

*N-(2-(4-(3-(Dimethylamino)propylamino)quinazolin-2-yl)phenyl)-2-morpholinoacetamide* (**10e**). Compound **7** was treated with excess morpholine according to the general aminolysis procedure to afford **10e** as a white solid in 78% yield after column chromatography with petroleum ether/EtOAc (20:1) elution; mp 179–181 °C. ^1^H-NMR (CDCl_3_) *δ =* 13.67 (s, 1H), 9.09 (s, 1H), 8.88 (d, *J* = 2.1 Hz, 1H), 8.53 (d, *J* = 8.6 Hz, 1H), 7.92 (d, *J* = 8.2 Hz, 1H), 7.77–7.69 (m, 1H), 7.62 (d, *J* = 8.1 Hz, 1H), 7.46 (t, *J* = 7.6 Hz, 1H), 7.13 (dd, *J* = 8.6, 2.2 Hz, 1H), 3.86 (dd, *J* = 10.2, 5.6 Hz, 2H), 3.64–3.53 (m, 4H), 3.32 (s, 2H), 2.68–2.62 (m, 2H), 2.63–2.53 (m, 4H), 2.42 (s, 6H), 1.94–1.85 (m, 2H). ^13^C-NMR (CDCl_3_) *δ =* 168.97, 160.56, 159.40, 148.43, 140.04, 136.45, 132.35, 131.83, 128.02, 126.02, 123.51, 122.86, 121.22, 120.55, 113.89, 66.34, 64.81, 59.95, 53.78, 45.51, 42.80, 24.41. HRMS (ESI): Calcd for (M−H)^− ^(C_25_H_31_ClN_6_O_2_) requires *m/z* 481.2119, found 481.2107. Anal. Calcd for C_25_H_31_ClN_6_O_2_: C, 62.17; H, 6.47; N, 17.40. Found: C, 62.24; H, 6.36; N, 17.55.

*N-(2-(4-(3-(Dimethylamino)propylamino)quinazolin-2-yl)phenyl)-3-(piperidin-1-yl)propanamide* (**11a**). Compound **8** was treated with excess piperidine according to the general aminolysis procedure to afford **11a** as a white solid in 69% yield after column chromatography with petroleum ether/EtOAc (15:1) elution; mp 178–179 °C. ^1^H-NMR (CDCl_3_) *δ =* 14.06 (s, 1H), 9.06 (s, 1H), 8.85 (d, *J* = 2.0 Hz, 1H), 8.66 (d, *J* = 8.6 Hz, 1H), 7.79–7.68 (m, 2H), 7.59 (d, *J* = 8.1 Hz, 1H), 7.48–7.40 (m, 1H), 7.08 (dd, *J* = 8.6, 2.1 Hz, 1H), 3.84 (dd, *J* = 10.2, 5.5 Hz, 2H), 2.92–2.82 (m, 2H), 2.80–2.71 (m, 2H), 2.67–2.58 (m, 2H), 2.54–2.42 (m, 4H), 2.40 (s, 6H), 1.96–1.85 (m, 2H), 1.61–1.53 (m, 4H), 1.46–1.37 (m, 2H). ^13^C-NMR (CDCl_3_) *δ =* 170.93, 160.60, 159.11, 148.02, 141.16, 136.75, 132.58, 131.74, 127.45, 125.93, 122.33, 121.79, 121.21, 120.01, 113.86, 59.94, 54.99, 54.42, 45.51, 42.83, 36.52, 25.97, 24.39, 24.30. HRMS (ESI): Calcd for (M−H)^− ^(C_27_H_35_ClN_6_O) requires *m/z* 493.2483, found 493.2472. Anal. Calcd for C_27_H_35_ClN_6_O: C, 65.51; H, 7.13; N, 16.98. Found: C, 65.70; H, 7.24; N, 16.86.

*N-(2-(4-(3-(Dimethylamino)propylamino)quinazolin-2-yl)phenyl)-3-(pyrrolidin-1-yl)propanamide* (**11b**). Compound **8** was treated with excess pyrrolidine according to the general aminolysis procedure to afford **11b** as a white solid in 71% yield after column chromatography with petroleum ether/EtOAc (15:1) elution; mp 163–165 °C ^1^H-NMR (CDCl_3_) *δ =* 14.12 (s, 1H), 9.07 (s, 1H), 8.86 (d, *J* = 2.0 Hz, 1H), 8.67 (d, *J* = 8.6 Hz, 1H), 7.74 (dt, *J* = 14.9, 7.6 Hz, 2H), 7.60 (d, *J* = 8.1 Hz, 1H), 7.45 (dd, *J* = 11.0, 3.9 Hz, 1H), 7.10 (dd, *J* = 8.6, 2.1 Hz, 1H), 3.87 (dd, *J* = 10.2, 5.5 Hz, 2H), 3.01 (t, *J* = 7.5 Hz, 2H), 2.82 (t, *J* = 7.5 Hz, 2H), 2.74–2.55 (m, 6H), 2.41 (s, 6H), 1.97–1.88 (m, 2H), 1.86–1.74 (m, 4H).^ 13^C-NMR (CDCl_3_) *δ =* 170.62, 160.68, 159.16, 148.07, 141.15, 136.84, 132.64, 131.73, 127.50, 125.94, 122.39, 121.81, 121.19, 120.09, 113.88, 59.96, 54.18, 52.08, 45.51, 42.84, 38.41, 24.40, 23.52. HRMS (ESI): Calcd for (M−H)^− ^(C_26_H_33_ClN_6_O) requires *m/z* 479.2326, found 479.2318. Anal. Calcd for C_26_H_33_ClN_6_O: C, 64.92; H, 6.91; N, 17.47. Found: C, 65.02; H, 6.68; N, 17.55.

*N-(2-(4-(3-(Dimethylamino)propylamino)quinazolin-2-yl)phenyl)-3-(diethylamino)propanamide* (**11c**). Compound **8** was treated with excess diethylamine according to the general aminolysis procedure to afford **11c** as a white solid in 60% yield after column chromatography with petroleum ether/EtOAc (15:1) elution; mp 158–160 °C. ^1^H-NMR (CDCl_3_) *δ =* 14.08 (s, 1H), 9.07 (s, 1H), 8.86 (d, *J* = 2.1 Hz, 1H), 8.67 (d, *J* = 8.6 Hz, 1H), 7.77–7.68 (m, 2H), 7.59 (d, *J* = 8.1 Hz, 1H), 7.44 (ddd, *J* = 8.1, 6.1, 2.0 Hz, 1H), 7.09 (dd, *J* = 8.6, 2.2 Hz, 1H), 3.86 (dd, *J* = 10.2, 5.6 Hz, 2H), 3.07–2.95 (m, 2H), 2.76–2.68 (m, 2H), 2.64 (t, *J* = 4.1 Hz, 2H), 2.63–2.56 (m, 4H), 2.41 (s, 6H), 1.97–1.85 (m, 2H), 1.05 (t, *J* = 7.1 Hz, 6H).^ 13^C-NMR (CDCl_3_) *δ =* 171.05, 160.60, 159.10, 147.99, 141.19, 136.74, 132.56, 131.74, 127.35, 125.93, 122.31, 121.74, 121.24, 119.95, 113.87, 59.90, 48.93, 47.01, 45.49, 42.73, 36.64, 24.41, 11.90. HRMS (ESI): Calcd for (M−H)^− ^(C_26_H_35_ClN_6_O) requires *m/z* 481.2483, found 481.2472. Anal. Calcd for C_26_H_35_ClN_6_O^.^H_2_O: C, 62.32; H, 7.44; N, 16.77. Found: C, 62.15; H, 7.57; N, 16.63.

*N-(2-(4-(3-(Dimethylamino)propylamino)quinazolin-2-yl)phenyl)-3-(4-methylpiperazin-1-yl)propane-mide* (**11d**). Compound **8** was treated with excess N-methylpiperazine according to the general aminolysis procedure to afford **11d** as a white solid in 44% yield after column chromatography with petroleum ether/EtOAc (10:1) elution; mp 167–168 °C. ^1^H-NMR (CDCl_3_) *δ =* 14.07 (s, 1H), 9.07 (s, 1H), 8.85 (d, *J* = 1.5 Hz, 1H), 8.66 (d, *J* = 8.6 Hz, 1H), 7.72 (d, *J* = 3.9 Hz, 2H), 7.60 (d, *J* = 8.1 Hz, 1H), 7.45 (dt, *J* = 8.1, 4.0 Hz, 1H), 7.09 (dd, *J* = 8.6, 1.8 Hz, 1H), 3.86 (dd, *J* = 10.2, 5.3 Hz, 2H), 2.92 (t, *J* = 7.4 Hz, 2H), 2.75 (t, *J* = 7.4 Hz, 2H), 2.67–2.63 (m, 2H), 2.59 (dd, *J* = 19.2, 11.0 Hz, 4H), 2.49–2.41 (m, 4H), 2.41 (s, 6H), 2.26 (s, 3H), 1.95–1.87 (m, 2H).^ 13^C-NMR (CDCl_3_) *δ =* 170.62, 160.71, 159.19, 148.08, 141.12, 136.87, 132.69, 131.77, 127.40, 125.98, 122.46, 121.85, 121.28, 120.12, 113.92, 59.92, 55.08, 54.20, 52.99, 46.01, 45.51, 42.80, 36.39, 24.42. HRMS (ESI): Calcd for (M−H)^− ^(C_27_H_36_ClN_7_O) requires *m/z* 508.2592, found 508.2588. Anal. Calcd for C_27_H_36_ClN_7_O: C, 63.58; H, 7.11; N, 19.22. Found: C, 63.49; H, 7.26; N, 19.36.

*N-(2-(4-(3-(Dimethylamino)propylamino)quinazolin-2-yl)phenyl)-3-morpholinopropanamide* (**11e**). Compound **8** was treated with excess morpholine according to the general aminolysis procedure to afford (**11e**) as a white solid in 67% yield after column chromatography with petroleum ether/EtOAc (15:1) elution; mp 165–167 °C. ^1^H-NMR (CDCl_3_) *δ =* 14.10 (s, 1H), 9.08 (s, 1H), 8.84 (d, *J* = 2.0 Hz, 1H), 8.67 (d, *J* = 8.6 Hz, 1H), 7.75–7.69 (m, 2H), 7.62 (d, *J* = 8.1 Hz, 1H), 7.45 (ddd, *J* = 8.2, 5.2, 2.9 Hz, 1H), 7.10 (dd, *J* = 8.6, 2.1 Hz, 1H), 3.86 (dd, *J* = 10.2, 5.4 Hz, 2H), 3.69–3.61 (m, 4H), 2.89 (t, *J* = 7.1 Hz, 2H), 2.75 (t, *J* = 7.2 Hz, 2H), 2.68–2.62 (m, 2H), 2.58–2.48 (m, 4H), 2.42 (s, 6H), 1.95–1.89 (m, 2H).^ 13^C-NMR (CDCl_3_) *δ =* 170.52, 160.59, 159.12, 147.94, 141.04, 136.77, 132.63, 131.77, 127.20, 125.99, 122.46, 121.80, 121.34, 120.02, 113.88, 66.88, 59.72, 54.61, 53.53, 45.46, 42.61, 36.28, 24.44. HRMS (ESI): Calcd for (M−H)^− ^(C_26_H_33_ClN_6_O_2_) requires *m/z* 495.2275, found 495.2271. Anal. Calcd for C_26_H_33_Cl N_6_O_2_^.^H_2_O: C, 60.63; H, 6.85; N, 16.32. Found: C, 60.51; H, 6.92; N, 16.36.

*N-(2-(4-(3-(Dimethylamino)propylamino)quinazolin-2-yl)phenyl)-4-(piperidin-1-yl)butanamide* (**12a**). Compound **9** was treated with excess piperidine according to the general aminolysis procedure to afford **12a** as a white solid in 73% yield after column chromatography with petroleum ether/EtOAc (15:1) elution; mp 159–160 °C. ^1^H-NMR (CDCl_3_) *δ =* 14.04 (s, 1H), 9.03 (s, 1H), 8.86 (d, *J* = 2.2 Hz, 1H), 8.68 (d, *J* = 8.6 Hz, 1H), 7.80–7.70 (m, 2H), 7.61 (d, *J* = 8.0 Hz, 1H), 7.45 (ddd, *J* = 8.2, 6.2, 2.0 Hz, 1H), 7.10 (dd, *J* = 8.6, 2.2 Hz, 1H), 3.87 (dd, *J* = 10.2, 5.7 Hz, 2H), 2.69–2.62 (m, 2H), 2.59 (t, *J* = 7.3 Hz, 2H), 2.53–2.46 (m, 2H), 2.46–2.41 (m, 4H), 2.41 (s, 6H), 2.04 (dt, *J* = 14.8, 7.4 Hz, 2H), 1.92 (dt, *J* = 11.3, 5.8 Hz, 2H), 1.56 (dt, *J* = 11.0, 5.5 Hz, 4H), 1.45–1.36 (m, 2H).^ 13^C-NMR (CDCl_3_) *δ =* 171.94, 160.74, 159.20, 148.17, 141.29, 136.90, 132.64, 131.77, 127.54, 125.94, 122.33, 121.81, 121.23, 120.05, 113.92, 60.00, 58.70, 54.56, 45.53, 42.86, 36.90, 25.95, 24.45, 22.94. HRMS (ESI): Calcd for (M−H)^− ^(C_28_H_37_ClN_6_O) requires *m/z* 507.2639, found 507.2629. Anal. Calcd for C_28_H_37_ClN_6_O: C, 66.06; H, 7.33; N, 16.51. Found: C, 66.10; H, 7.49; N, 16.32.

*N-(2-(4-(3-(Dimethylamino)propylamino)quinazolin-2-yl)phenyl)-4-(pyrrolidin-1-yl)propanamide* (**12b**). Compound **9** was treated with excess pyrrolidine according to the general aminolysis procedure to afford **12b** as a white solid in 66% yield after column chromatography with petroleum ether/EtOAc (15:1) elution; mp 162–164 °C. ^1^H-NMR (CDCl_3_) δ 13.90 (s, 1H), 8.97 (s, 1H), 8.81–8.65 (m, 2H), 7.78–7.69 (m, 2H), 7.61 (d, *J* = 8.0 Hz, 1H), 7.47–7.40 (m, 2H), 7.17–7.12 (m, 1H), 3.88 (dd, *J* = 10.2, 5.7 Hz, 2H), 2.62 (dd, *J* = 13.8, 6.6 Hz, 3H), 2.54 (s, 2H), 2.40 (s, 3H), 2.11–2.02 (m, 2H), 1.95–1.88 (m, 2H), 1.80–1.71 (m, 2H). ^13^C-NMR (CDCl_3_) δ 171.76, 161.30, 159.18, 148.24, 140.35, 132.45, 130.95, 130.69, 127.42, 125.75, 123.68, 122.26, 121.25, 120.26, 113.77, 59.59, 55.83, 54.07, 45.45, 42.40, 36.92, 25.19, 24.68, 23.45. HRMS (ESI): Calcd for (M−H)^−^ (C_28_H_37_ClN_6_O) requires *m/z* 507.2639, found 507.2629. Anal. Calcd for C_28_H_37_ClN_6_O: C, 66.06; H, 7.33; N, 16.51. Found: C, 66.10; H, 7.49; N, 16.32.

*N-(2-(4-(3-(Dimethylamino)propylamino)quinazolin-2-yl)phenyl)-4-(diethylamino)propanamide* (**12c**). Compound **9** was treated with excess diethylamine according to the general aminolysis procedure to afford **12c** as a white solid in 63% yield after column chromatography with petroleum ether/EtOAc (15:1) elution; mp 159–161 °C. ^1^H-NMR (CDCl_3_) δ 13.84 (s, 1H), 9.06 (s, 1H), 8.65 (dd, *J* = 12.9, 4.9 Hz, 2H), 7.71–7.61 (m, 3H), 7.36 (dd, *J* = 11.1, 4.2 Hz, 2H), 7.12–7.03 (m, 1H), 3.81 (dd, *J* = 9.9, 5.4 Hz, 2H), 2.99–2.90 (m, 2H), 2.69–2.59 (m, 8H), 2.54 (q, *J* = 7.1 Hz, 4H), 1.90–1.81 (m, 2H), 1.07 (t, *J* = 7.1 Hz, 6H), 0.98 (t, *J* = 7.1 Hz, 6H).^ 13^C-NMR (101 MHz, CDCl3) δ 170.79, 161.43, 159.14, 148.27, 140.27, 132.51, 131.02, 130.64, 127.49, 125.55, 123.73, 122.34, 121.59, 120.34, 113.78 , 53.54, 48.96, 46.98, 42.97, 36.53, 24.23, 11.43. HRMS (ESI): Cacld for (M-H)^− ^(C_28_H_38_N_6_O) requires m/z 475.3185, found 475.3186.

*N-(2-(4-(3-(Dimethylamino)propylamino)quinazolin-2-yl)phenyl)-4-(4-methylpiperazin-1-yl)butanami-de* **12d**. The compound **9** was treated with excess *N*-methyl piperazine according to general aminolysis procedure to afford **12d** as a white solid in 58% yield after column chromatography with petroleum ether/EtOAc (10:1) elution; mp 155–157 °C. ^1^H-NMR (CDCl_3_) *δ =* 14.03 (s, 1H), 9.02 (s, 1H), 8.85 (d, *J* = 2.1 Hz, 1H), 8.66 (d, *J* = 8.7 Hz, 1H), 7.72 (dd, *J* = 3.9, 1.6 Hz, 2H), 7.60 (d, *J* = 8.2 Hz, 1H), 7.43 (ddd, *J* = 8.2, 5.4, 2.8 Hz, 1H), 7.08 (dd, *J* = 8.7, 2.2 Hz, 1H), 3.89 (dd, *J* = 10.2, 5.5 Hz, 2H), 2.75–2.69 (m, 2H), 2.66 (t, *J* = 7.3 Hz, 2H), 2.59 (t, *J* = 7.3 Hz, 2H), 2.55–2.46 (m, 4H), 2.43 (s, 6H), 2.36–2.28 (m, 4H), 2.23 (s, 3H), 2.02 (dt, *J* = 14.5, 7.3 Hz, 2H), 1.97–1.90 (m, 2H).^ 13^C-NMR (CDCl_3_) *δ =* 171.86, 160.64, 159.12, 148.05, 141.26, 136.80, 132.60, 131.75, 127.42, 125.91, 122.26, 121.71, 121.26, 119.95, 113.89, 59.89, 57.81, 55.10, 53.11, 45.99, 45.49, 42.76, 36.75, 24.41, 22.93. HRMS (ESI): Calcd for (M−H)^− ^ (C_28_H_38_ClN_7_O) requires *m/z* 522.2748, found 522.2747. Anal. Calcd for C_28_H_38_ClN_7_O: C, 64.17; H, 7.31; N, 18.71. Found: C, 64.03; H, 7.26; N, 18.59.

*N-(2-(4-(3-(Dimethylamino)propylamino)quinazolin-2-yl)phenyl)-4-morpholinobutanamide* (**12e**). Compound **9** was treated with excess morpholine according to the general aminolysis procedure to afford **12e** as a white solid in 65% yield after column chromatography with petroleum ether/EtOAc (15:1) elution; mp 152–154 °C. ^1^H-NMR (CDCl_3_) *δ =* 14.07 (s, 1H), 9.03 (s, 1H), 8.87 (d, *J* = 2.2 Hz, 1H), 8.68 (d, *J* = 8.6 Hz, 1H), 7.78–7.69 (m, 2H), 7.65 (d, *J* = 8.2 Hz, 1H), 7.46 (ddd, *J* = 8.2, 5.0, 3.2 Hz, 1H), 7.10 (dd, *J* = 8.6, 2.2 Hz, 1H), 3.88 (dd, *J* = 10.3, 5.6 Hz, 2H), 3.70–3.56 (m, 4H), 2.73–2.64 (m, 2H), 2.61 (t, *J* = 7.2 Hz, 2H), 2.47 (t, *J* = 6.3 Hz, 2H), 2.44 (s, 6H), 2.08–1.99 (m, 2H), 1.98–1.91 (m, 2H), 1.86–1.72 (m, 4H).^ 13^C-NMR (CDCl_3_) *δ =* 171.82, 160.65, 159.12, 148.02, 141.24, 136.83, 132.60, 131.77, 127.30, 125.96, 122.32, 121.68, 121.32, 119.93, 113.89, 66.97, 59.81, 58.27, 53.67, 45.48, 42.68, 36.62, 24.43, 22.52. HRMS (ESI): Calcd for (M−H)^− ^(C_27_H_35_ClN_6_O_2_) requires *m/z* 509.2432, found 509.2429. Anal. Calcd for C_27_H_35_ClN_6_O_2_: C, 63.45; H, 6.90; N, 16.44. Found: C, 63.31; H, 7.05; N, 16.51.

### 3.2. Materials and Biological Assays

Stock solutions of all quinazoline derivatives (10 mM) were made with DMSO and stored at 4 °C. All tumor cell lines were obtained from the American Type Culture Collection (ATCC, Rockville, MD, USA). The cell culture was maintained in a RPMI-1640 or DMEM medium supplemented with 10% fetal bovine serum, 100 U/mL penicillin, and 100 mg/mL streptomycin in 25 cm^2^ culture flasks at 37 °C humidified atmosphere with 5% CO_2_. Chick embryos were purchased from South China Agricultural University (SCAU).

#### 3.2.1. Methyl Thiazolyl Tetrazolium (MTT) Assay

HUVEC, CNE-2, PC-3, and 7721 cell lines were seeded on 96-well plates at a concentration of 5,000 cells per well and exposed to various concentrations of quinazoline derivatives. After 48 h of treatment at 37 °C in a humidified atmosphere of 5% CO_2_, 5 mg/mL MTT solution (10 μL) was added to each well and further incubated for 4 h. Each well was then added dimethyl sulfoxide (DMSO, 100 μL), and the optical density (OD) was recorded at 490 nm. All measurements were done in triplicate, and the IC_50_ values were derived from the mean OD values of the triplicate tests versus drug concentration curves [12,13].

#### 3.2.2. Cell Adhesion Assay

Cell adhesion assay was carried out by a reported method [14,15] with slight modification. Wells of a 96-well plate were coated at room temperature overnight with rat trail tendon collagen type I (2 μg) in PBS in a final volume of 100 μL. The wells were washed three times with PBS (100 mL) and then blocked with 0.2% bovine serum albumin (BSA) in PBS (100 mL) for 2 h at 37 °C in a cell culture incubator. The wells were then washed three times with PBS (100 mL). HUEVCs were detached from the dish, resuspended in DMEM, and added (50 μL, 10^4 ^cells/mL) to each well followed by the related compounds (50 μL) at various concentrations. The plates were incubated for 1 h or 3 h at 37 °C and then washed three times with PBS (100 μL). The attached cells were fixed and stained with 0.2 % crystal violet in 20% methanol for 10 min, then washed three times with PBS (100 mL). The cells were solubilized by 2% SDS and the OD was measured at 570 nm. Attachment was determined by both 1 and 3 h as described above. Each compound was tested in triplicate and each experiment was repeated three times.

#### 3.2.3. Cell Migration Assay

The HUVEC cell line was seeded on a pre-treated 48-well culture plate at a concentration of 2 × 10^4^ cells per well in a volume of 200 μL, and a portion of the cell monolayer was scraped off with a sterile disposable rubber policeman [15,16,17]. After the DMEM medium was removed, the cells in each well were then washed three times with PBS (phosphate-buffer saline), and fresh medium was added to each well. The wells were then tested after incubation for 24 h with or without quinazoline derivatives at various concentrations. Two random pictures were taken for each wound using a microscope. The area of the migrations was measured using Photoshop CS_3_. All measurements were done in triplicate, and the experiments were done at least three times.

#### 3.2.4. CAM Assay

The *in vivo* CAM angiogenesis model as initially described by Folkman [10] and then modified by Maragoudakis [18,19,20,21] was used. Fertilized chicken eggs were used after incubating them for 8 days. A 1 cm × 1 cm window was made in the shell to create a pocket to expose the CAM. A filter disc with 0.9% NaCl solution or 30 μg/10 μL of compounds was then placed upon the CAM from 10 eggs per treatment and 48 h later, the CAMs were fixed with 4% paraformaldehyde and the vessel number from three random fields around the filter disc were pictured using a microscope.

## 4. Conclusions

Tumor angiogenesis is a promising target of cancer therapy. Based on this target, a structure-based drug design plan led us to synthesize a series of 2,4-disubstituted quinazoline derivatives that were then subjected to pharmacological evaluation as angiogenesis inhibitors. Cytotoxicity assays indicated that most of 2,4-disubstituted quinazoline derivatives showed potent cytotoxicity against both tumor cells and HUVECs,and that their cytotoxicity toward tumor cells was closely related to the length of side chains and basicity. Among them, compound **11d** with a three carbon chain length and the N-methylpiperazino group showed a remarkable inhibitive effect against the migration, adhesion of HUVECs and significant anti-angiogenesis activities in the chick embryo chorioallantoic membrane (CAM) assay, which are consistent with those previously reported for effective angiogenesis inhibitors. Finally, this preliminary research on these quinazoline derivatives’ *in vitro* activities and *in vivo* activities provides us with another new entry into angiogenesis inhibitors and conformation studies for targeting tumor angiogenesis. Additional studies and tests on their mechanism of anti-angiogenesis activity are underway.
